# Emerging targeted therapies for melanoma treatment (Review)

**DOI:** 10.3892/ijo.2014.2481

**Published:** 2014-06-03

**Authors:** ANGELA RUSSO, BARTOLOMEA FICILI, SAVERIO CANDIDO, FRANCA MARIA PEZZINO, CLAUDIO GUARNERI, ANTONIO BIONDI, SALVATORE TRAVALI, JAMES A. McCUBREY, DEMETRIOS A. SPANDIDOS, MASSIMO LIBRA

**Affiliations:** 1 Laboratory of Translational Oncology and Functional Genomics, Section of General Pathology and Oncology, Department of Biomedical Sciences, University of Catania, I-95124 Catania, Italy; 2 Department of Social Territorial Medicine, Section of Dermatology, University of Messina, I-98125 Messina, Italy; 3 Department of Surgery, University of Catania, I-95124 Catania, Italy; 4 Department of Microbiology and Immunology, Brody School of Medicine at East Carolina University, Greenville, NC, USA; 5 Department of Virology, Medical School, University of Crete, Heraklion 71003, Crete, Greece

**Keywords:** melanoma, MAPK/AKT pathway, targeted therapies

## Abstract

Cutaneous melanoma is an aggressive cancer with a poor prognosis for patients with advanced disease. The identification of several key molecular pathways implicated in the pathogenesis of melanoma has led to the development of novel therapies for this devastating disease. In melanoma, both the Ras/Raf/MEK/ERK (MAPK) and the PI3K/AKT (AKT) signalling pathways are constitutively activated through multiple mechanisms. Targeting various effectors of these pathways with pharmacologic inhibitors may inhibit melanoma cell growth and angiogenesis. Ongoing clinical trials provide hope to improve progression-free survival of patients with advanced melanoma. This review summarizes the most relevant studies focused on the specific action of these new molecular targeted agents. Mechanisms of resistance to therapy are also discussed.

## 1. Introduction

Cutaneous melanoma, is a form of aggressive cancer that develops from melanocytes. It is most common in people between 30 and 60 years of age. The highest incidence rates occurs in white-skinned peoples living at low latitudes (1). Accordingly, the association between sun exposure and melanoma have been explored. An important risk factor for melanoma is UV irradiation upon sun exposure (2). Indication of a direct UV mutagenic effect in melanoma development remains controversial as the nucleotide exchange detected in the *B-RAF* gene (T/A) is not classically linked to UV mutagenesis signature attributable to cytidine to thymidine (C→T) transitions. As suggested, it is possible that *B-RAF* mutations could arise from error prone replication of UV-damaged DNA (3). Melanoma is a heterogeneous disease that presents different genetic alterations and variety of histologic subtypes (4). *B-RAF* mutations were commonly detected in cutaneous melanomas arising from intermittent sun-exposed sites (5). Accordingly, we have, recently, identified a higher frequency of *B-RAF*^V600E^ mutation in melanoma of the trunk from indoor workers compared to outdoor workers, suggesting that this mutation may be associated with an intermittent exposure to the sun, as usually the trunk is a sun-protected body site (6).

**Figure 1:**
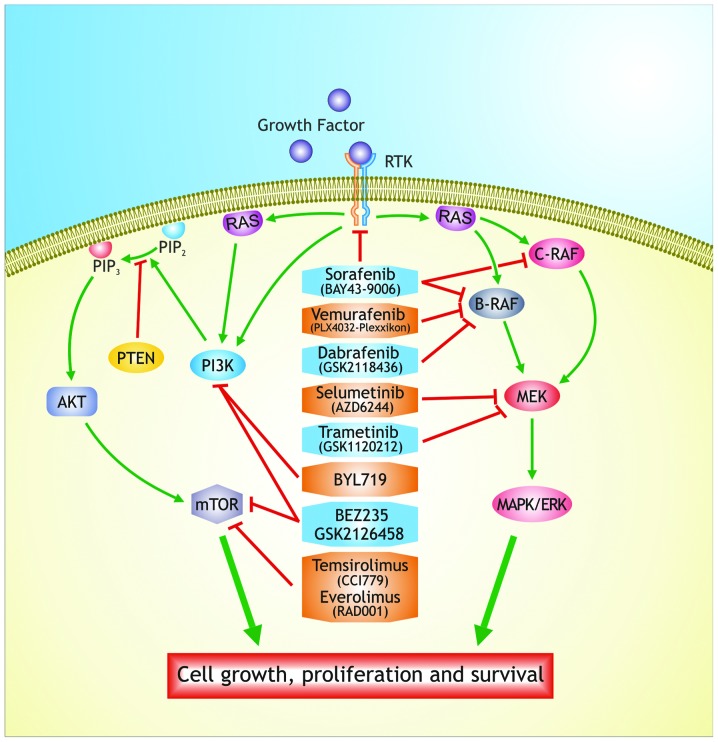
Raf/MEK/ERK and PI3K/AKT pathways and mechanism of action of their inhibitors in melanoma.

Melanomas exhibit mutations in the Ras/Raf/mitogen activated protein kinase (MAPK) pathway. Over 50% of melanomas harbor activating mutations in *B-RAF* gene (*B-RAF*^V600E^) (7,8), known to play a key role in proliferation and survival of melanoma cells through activation of the MAPK pathway (9). Furthermore, this mutation causes constitutive activation of the kinase as well as insensitivity to negative feedback mechanism (10). *B-RAF*^V600E^, *B-RAF*^V600K^, *B-RAF*^V600R^ and *B-RAF*^V600D^ mutations were detected at the frequency from 6 to 3% (7). Knowledge on the deregulation of MAPK and P3K pathways in different cancer types, including melanoma, has led to the development of specific inhibitors of their key components (11–14) (Fig. 1).

A list of current clinical trials for melanoma is available on the NCI Web site (http://www.cancer.gov/clinicaltrials). For stage IV (TNM) melanoma we found a total of 281 ongoing clinical trials. Most of these studies are evaluating whether any benefits are observed after treatment with novel combination therapies or tailored therapies compared to standard treatments. In Table I the MAPK/AKT inhibitors with their targets are summarized. The prognosis for melanoma patients at early stage of the disease is 90% survival by surgical treatment. In contrast, the prognosis for advanced melanoma is restricted due to the development of drug resistance after treatment with chemotherapeutic agents (15). This review is focused on the clinical application for the treatment of melanoma with MAPK and AKT inhibitors and other novel therapies. Mechanisms of resistance for each therapy are also discussed.

## 2. MAPK/MEK/ERK inhibitors

### Sorafenib

Sorafenib (BAY43-9006, Nexavar, Bayer Pharmaceuticals Corp., West Haven, CT, USA) is a potent multi-kinase inhibitor that targets also the receptor tyrosine kinase-associated angiogenesis (VEGFR-2, VEGFR-3, PDGF-β) and tumor progression (c-KIT, FLT-3) (16,17). Sorafenib was initially developed as an inhibitor of the RAF serine/threonine kinases and administered orally in combination with carboplatin and taxol in patients with lung cancer (10).

The study of this inhibitor in xenograft models showed that sorafenib inhibited tumor cell proliferation and/or endothelial cell mediated tumor angiogenesis in several forms of human cancer (18). Sorafenib administered as monotherapy has a manageable side effect profile in phase I/II/III studies (19–21) and the most common toxic effects are hand-foot skin reaction (HFS), rash and diarrhoea (22). Sorafenib is effective in the treatment of a small percentage of melanomas that carry mutations G469E and D594G in *B-RAF* gene expressing constitutively ERK1/2, low levels of MEK. However, it did not show significant benefit in melanoma patients harboring *B-RAF*^V600E^ mutation (23). It was suggested that a receptor kinase upstream of Ras/Raf/MEK/ERK cascade may be targeted by sorafenib (24). This inhibitor may be administered in combination with an inhibitor of MEK in the treatment of more aggressive forms of melanoma. It may target the VEGF and other membrane receptors expressed in cancer cells, whereas the MEK inhibitor blocks the cascade which is abnormally activated by *B-RAF* (25). The sorafenib dose (400 mg b.i.d) is administered in combination with standard chemotherapy, such as dacarbazine, in patients with advanced melanoma because it has few side effects as a single agent, indeed the response rate was 21% with a median time from treatment initiation of 2.3 months (26). Although this combination does not cause toxic effects and shows antitumor activity, it is not applied in clinical practice because selective inhibitors of *B-RAF* are more effective in the treatment of malignant melanoma (27).

### Vemurafenib

Vemurafenib (Zelboraf, Plexxikon/Roche) was approved first by the FDA in USA, in August 2011, for the treatment of patients with metastatic melanoma with *B-RAF*^V600E^ mutation and then in Europe (15). Vemurafenib (PLX4032) is a potent oral drug that inhibits the kinase domain of the most common mutation of *B-RAF* (*B-RAF*^V600E^), decreasing cell proliferation through the phosphorylation of ERK and cyclin D1 (28,29), but it does not have antitumor effects against cells with *B-RAF*^WT^ (30,31).

The pharmacodynamic analysis reported that the activity of vemurafenib was characterized as exposure-dependent tumor response corresponding with percentage of inhibition of MEK and ERK phosphorylation. Additionally, the relationship between dose exposure and response suggests that melanoma regression was found to correlate with >90% inhibition of ERK phosphorylation (32).

Patients with advanced melanoma and *B-RAF* mutations showed in phase I and II clinical trials of vemurafenib an antitumor response in more than 50% of the patients. A phase III study comparing vemurafenib with dacarbazine in previously untreated patients revealed an overall survival rate of 84% among patients treated with vemurafenib and 64% in the other group of patients. Vemurafenib was associated with a relative reduction of 63% in the risk of death and 74% in the risk of either death or disease progression, as compared with dacarbazine (30). The maximum tolerated dose is 960 mg twice daily, showing positive tumor responses. Patients who had received previous treatment for melanoma with *B-RAF*^V600E^ mutation showed a response rate of 53%, with a median duration of response of 6.7 months (33). In addition, vemurafenib causes acanthopapillomas, keratoacanthomas and cutaneous squamous cell carcinomas in the early treatment (34,35). Vemurafenib demonstrates an exceptional response in melanoma patients with *B-RAF*^V600E^ mutation and its introduction represented a step forward in the treatment of this disease (36). Despite the encouraging results obtained, the duration of response is limited because tumors quickly develop resistance via molecular alterations in other pathway components (37). Drug resistance is a common problem associated with treatment with chemotherapeutic agents. To evaluate the mechanisms of resistance is essential to understand the adaptability of tumor cells and the multiple mechanisms that lead to drug resistance (14,38). Resistance mechanisms can be divided into MAPK-dependent and MAPK-independent pathways.

MAPK-dependent resistance mechanisms lead to reactivation of ERK, changes in *B-RAF*, such as amplification of mutant *B-RAF* or truncations in the B-Raf protein through alternate splicing leading to increased dimerization and resultant kinase activity (39). In addition, resistance is caused by secondary mutations in the MAPK pathway immediately upstream at the level of *N-RAS* and downstream at the level of MEK, which render the kinase insensitive to the inhibitor (40).

MEK1/2 are phosphorylated and activated by *B-RAF*; mutation in *MEK1* (P124L) was identified to be responsible for cellular resistance to PLX 4032 (41). The resistance to treatment occurs after an initial response (42). The potent antitumor effect of vemurafenib is mediated through inhibition of the oncogenic MAPK signaling. Clinical trials are currently underway in the treatment of advanced melanoma to test the efficacy of vemurafenib with immunomodulatory agents, such as ipilimumab, and in combination with MEK inhibitors, such as GDC-0973 (43).

### Dabrafenib

Dabrafenib (GSK2118436) is a reversible ATP-competitive inhibitor that selectively inhibits *B-RAF*. It is similar to vemurafenib concerning the mechanism of action, pharmacodynamics, timing of responses and development of resistance, but it presents a shorter half-life (44). Dabrafenib is efficient in about 50–70% of patients with *B-RAF*^V600E^ or *B-RAF*^V600K^ mutations (45,46).

A phase I/II study of dabrafenib established a dose of 150 mg twice daily and reported positive responses in about 50% of the patients with advanced melanoma and a median progression-free survival of 6-3 months (47). The most common cutaneous side effects were hyperkeratosis, papillomas and palmar-plantar erythrodysaesthesia; other side effects were pyrexia, fatigue, headache and arthralgia, which together necessitated dose reductions (48).

In a phase III study, comparing dabrafenib vs. dacarbazine, it has been demonstrated that the response rates were 53 vs. 19% and PFS 5.3 vs. 2.7, although the trial allowed crossover and was not powered to detect an overall survival benefit (49,50). The low rate of survival benefit was partly due to the obligatory crossover to dabrafenib at progression in patients randomly assigned to dacarbazine (29).

### MEK inhibitors

MEK proteins belong to a family of enzymes, that selectively phosphorylate serine/threonine and tyrosine residues within the activation loop of their specific MAP kinase substrates. MEK1 and MEK2 display a similar structural organization, are closely related and they participate in the Ras/Raf/MEK/ERK signal transduction cascade (51,52).

Several MEK inhibitors have been tested in clinical trials. Selumetinib (AZD6244), is an oral small molecule that inhibits MEK1/2 and has been tested clinically in a randomize phase II trial in patients with *B-RAF* mutated melanoma (53). In a phase III study, only trametinib (known as GSK1120212 or JTP-74057), a selective oral inhibitor of MEK1 and 2, has been demonstrated to have impact on clinical efficacy (54,55). Trametenib causes a block of the protein MEK, and is correlated with improved PFS in patients carrying *B-RAF*^V600E/K^ mutations (56).

Previous studies showed that trametinib inhibits cell growth by the inhibition of pERK 1/2, inducing cell cycle arrest in cell lines with mutant *B-RAF* and *RAS*. This shows its potent antitumor activity when administered daily for 14 days (57–59). In a phase III trial (METRIC), trametinib was compared with dacarbazine in patients with *B-RAF* mutations (60), observing an improvement in median survival of 81 vs. 67% and PFS of 4.8 vs. 1.5 months, with an objective response rate about 25% (61). Administration of trametinib, as monotherapy, results in a low activity in patients previously treated with B-RAF inhibitors. Resistance to B-RAF inhibitors may be also associated with resistance to MEK inhibitors. In patients treated with trametinib the most common toxic effects included skin rash, diarrhea, edema, hypertension and fatigue (62). Trametinib compared with chemotherapy showed a significant improvement in progression-free and overall survival in patients with advanced and/or metastatic melanoma (60).

## 3. PI3K/AKT/mTOR inhibitors

PI3K/AKT/mTOR pathway is one of the most frequently dysregulated pathway in human cancer. The most frequent causes of changes in this pathway include mutation or increased gene copy numbers of *PIK3CA* or other PI3K isoforms, loss of expression of the pathway suppressors (for example, PTEN) or hyperactivation of RTKs through receptor overexpression or activating mutations (63–66). Hot spot mutations of the *PIK3CA* gene include *E542K, E545K* and *H1047R*. These mutations are oncogenic *per se*, as they can induce the generation of tumors in several preclinical models without other molecular aberrations (67–69).

The PI3K/AKT/mTOR and RAS/RAF/MEK/ERK pathways interact at multiple points, resulting in cross-activation, cross-inhibition, pathway convergence and these observations have driven the development of small molecule inhibitors that target various nodes of both pathways (70).

Recent studies have revealed that PI3K signalling is deregulated in a high proportion of melanomas (11). Indeed, *PTEN* is deleted and the downstream *AKT* gene is amplified in about 45% of melanomas. These alterations cause an overexpression of AKT3, an isoform of AKT (71). Increased phospho-AKT expression in melanoma is associated with tumor progression and shorter survival (72). The study of genomic alterations in primary melanomas showed that tumors with *B-RAF* mutations had few copies of *PTEN*, suggesting that dual activation of the PI3K-AKT and MAPK pathways are important events in melanoma development (73).

The research on PI3K inhibitors is expanding in order to find more selective compounds, such as isoform-specific PI3K inhibitors (74).

Advanced studies led to the development of inhibitors of PI3K which selectively target only the catalytic sites (75). The new PI3Ka isoform-specific inhibitors gave an effective response in cell lines that present *PIK3CA* mutations (76,77). In addition, one of these compounds, BYL719, has revealed that besides *PIK3CA* mutations, the presence of *PI3KCA* amplification correlated with higher drug sensitivity, while *B-RAF* and *PTEN* mutations were correlated with resistance (74).

The PI3K inhibitors, GSK2126458 and BEZ235, were evaluated *in vitro* in combination with MEK inhibitors, showing enhanced cell growth inhibition. Monotherapy with inhibitors of PI3K did not show advantage in clinical response, suggesting their use in combination with other drugs (78). The low efficacy of the monotherapy treatments is due to the interaction between the parallel PI3K/AKT/mTOR and RAS/RAF/MEK/ERK pathways and the resistance to therapy can be induced by overexpression or overactivation of PDGFR-β or IGF1R (25). To overcome the resistance mechanisms, dual inhibition of both pathways with combined therapy may be appropriate (14). PI3K and MEK inhibitor combinations are well tolerated and can be administered at therapeutic doses; however, additional studies are required to establish the precise tumor properties that will better respond to therapy (38). Rapamycin is a mTORC1 blocker of the first generation, while everolimus (RAD001) and temsirolimus (CCI779) are considered agents of second generation, which allosterically inhibit the mTOR complex (79,80); these agents do not have high specificity in targeting melanoma tumor cells (73).

## 4. Immunotherapy and ipilimumab

New therapeutic approaches involve the use of immunotherapy for the treatment of cancer. Immunotherapy is based on increasing the immune defenses to eliminate the cancer cells to gain chemotherapeutic effect, and aiming to arrest the cell cycle inducing apoptosis (81).

Immunotherapy may be used for tumors because they express tumor associated antigens (82,83); melanoma lesions often contain a high number of infiltrative T-cells specific to melanocyte tumor-associated antigens such as MART1, gp100 and tyrosinase (84). An approach to eliminate the melanoma cells is to increase the natural function of these cytotoxic T lymphocytes (CTL) (85).

Cytotoxic T lymphocyte-associated antigen 4 (CTLA-4) is an immunoglobin-like molecule found primarily on CD4^+^ or CD8^+^ T lymphocytes and high levels of CTLA-4 are also important in maintaining certain subsets of T-regulatory cells. CTLA-4 is an ‘immune checkpoint’ that downregulates pathways of T-cell activation and prevents autoimmunity, for this purpose it has become an attractive target for immunotherapy (86).

The first immunotherapy to be approved by the Food and Drug Administration (FDA) for treatment of advanced melanoma was interleukin-2 (IL-2) but, like dacarbazine, response rates were low even at high-doses of treatment (87). Its use in clinical practice is limited by the severe toxic side-effects (88–90).

Ipilimumab is a recombinant, human monoclonal antibody that binds to CTLA-4 and blocks the interaction of CTLA-4 with its ligands, CD80 and CD86. This immunotherapeutic augments the antitumor T-cell response resulting in uncontrolled T-cell proliferation and for this reason is associated with a substantial risk of immune-related adverse reactions (91,92). Ipilimumab acts by an indirect mechanism through T-cell mediated antitumor immune responses. The most common severe immune-mediated adverse reactions are enterocolitis, hepatitis, dermatitis, neuropathy and endocrinopathy; these reactions can occur both during the treatment, or weeks or months after discontinuation of treatment (93,94).

Ipilimumab was approved, as monotherapy at 3 mg/kg, in the European Union in 2011 for pretreated adult patients with advanced (unresectable or metastatic) melanoma and in the United States for both first- and second-line treatment for advanced melanoma (95,96). In a clinical study, untreated patients with advanced melanoma received a higher dose of ipilimumab with or without dacarbazine or dacarbazine plus placebo. Patients treated with dacarbazine and ipilimumab showed a significant increase of the overall survival rate compared with those treated with dacarbazine plus placebo (29). The treatment with ipilimumab in advanced melanoma patients was also considered in concomitance with the experimental vaccine glycoprotein 100 (gp100) (97). Patients with advanced melanoma stage III or IV were included in this study. Pre-treated patients were randomized for the administration of ipilimumab alone or in combination with gp100 or gp100 alone. It was shown that the combination with ipilimumab and gp100 did not improve survival when compared with ipilimumab alone, suggesting that ipilimumab remains the treatment with most efficacy for advanced melanoma (98).

Besides ipilimumab, also nivolumab, a monoclonal antibody directed against the PD-1 receptor or its ligand (PD-L1) has been reported (97). PD-1 receptor acts as an inhibitory receptor of T cells similar to CTLA-4. In an initial phase I study of a monoclonal antibody binding the PD-1 receptor, in patients previously treated, tumor responses were recorded in 26 of 94 (28%) (99). Nivolumab has been administered as monotherapy; most recent data suggest that nivolumab and ipilimumab can be administered concomitantly with a manageable safety profile (100). Immunotherapy is becoming an important support to melanoma treatment (101).

## 5. Combination therapy

Resistance to therapeutic agents, both chemical or biological agents, remains the main problem in the management of the therapy in melanoma. Combination of B-RAF inhibitors with MEK inhibitors has been evaluated to improve the disease-free survival. This combination reduces the skin toxicities and may also enhance the antitumoral effects by synergistically suppressing ERK pathways activity (102).

In patients who have developed resistance to vemurafenib, the combination of dabrafenib and trametinib showed 76% of clinical response compared with that obtained with the treatment of dabrafenib as single agent (54%) (41).

In a phase I/II trial, the combination of dabrafenib and trametinib was effective in patients with *B-RAF*^V600E^ mutated metastatic melanoma and numerous clinical trials are in progress to test other combinations of B-RAF and MEK inhibitors (103,104). In a phase I study the effectiveness of vemurafenib was tested in combination with an inhibitor of MEK showing a tumor reduction in melanoma patients, while in a phase III trial vemurafenib alone was compared with vemurafenib in combination with MEK inhibitor (36). Several clinical studies are still evaluating the combination of PI3K and MEK inhibitors in a variety of cancers. This combined therapy may be able to overcome the resistance mechanisms leading to apoptosis. These combinations appear well tolerated and can be administered as therapeutic doses (80).

The approval of ipilimumab represents a further treatment option for melanoma patients. The National Comprehensive Cancer Network (NCCN) now lists ipilimumab and vemurafenib among the small number of preferred systemic regimens for treating advanced and metastatic melanoma (105). The combination of vemurafenib with immunotherapy could overcome the resistance mechanisms because immunotherapy drugs have low response rates but relatively long durations of response in a large subset of responding patients, by contrast, B-RAF inhibitors have high initial response rates but rarely produce long-term durable responses (95,106). Ipilimumab targets the tumors indirectly by activation of the immune system therefore it is likely to be efficacious in melanoma patients with and without the *B-RAF*^V600E^ mutation (100).

Some clinical trials have shown that MAPK pathway inhibition with a selective inhibitor of *B-RAF*^V600E^ increase expression of melanoma-derived antigens by the tumor and increased the recognition of melanoma cells by antigen-specific T cells and, selective inhibition did not have deleterious effects on T cell proliferation or function (100,107).

B-RAF inhibitor treatment led to increased number of tumor infiltrating lymphocytes in tumor biopsies obtained 10–14 days after treatment initiation. This increase was associated with a reduction in tumor size and an increase in necrosis in on-treatment biopsies (108,109).

These results suggested that at least a subset of patients might be able to receive treatment with curative intent with interleukin-2 or ipilimumab without compromising their ability to benefit from B-RAF inhibitor treatment if they fail to achieve a durable response (106).

Studies that combine B-RAF inhibitors with immunotherapy are in progress and offer opportunities to further improve outcomes for patients with advanced B-RAF V600E mutant melanoma.

Finally, the effects of the new nitric oxide (NO) donating compound (S,R)-3-phenyl-4,5-dihydro-5-isoxazole acetic acid-nitric oxide (GIT-27NO) on the A375 human melanoma cell line were investigated by our group. The capacity of GIT-27NO to induce p53-mediated apoptosis in A375 melanoma cells suggests that GIT-27NO may have a potential therapeutic use in the clinical setting (110,111).

## 6. Conclusion

Cancer research is converging on understanding the roles of signal transduction pathways in drug resistance and sensitivity. Targeting various effectors of these pathways with pharmacologic inhibitors may arrest melanoma cell proliferation. This review pays attention on the clinical application of both Raf/MEK/ERK and PI3K/AKT/mTOR pathway inhibitors as novel treatment strategy for melanoma. Furthermore, we described how alterations of molecular pathways, involved in melanoma development, interact with each other resulting in response to therapy and/or chemoresistance. The use of MAPK and AKT inhibitors for the treatment of melanoma indicates that the response rate of these new molecular targeted agents is higher compared to the standard chemotherapy. However, additional studies are needed to better define the mechanisms of resistance to these novel biological therapies.

## Figures and Tables

**Table I:** MAPK/AKT inhibitors and their targets.

Drug	Target	Pathway
Sorafenib (BAY43-9006)	*B-RAF*, *C-RAF*, *VEGF-R*, *PDGF-R*	Ras/Raf/MEK/ERK
Vemurafenib (PLX-4032)	*B-RAF*^(V600E, V600K)^	
Dabrafenib (GSK 2118436)	*B-RAF*^(V600E, V600K)^	
Trametinib (GSK1120212)	MEK1/2	
Selumetinib (AZD6244)	MEK1/2	
BEZ235	PI3K-mTOR	PI3K/AKT/mTOR
GSK2126458	PI3K-mTOR	
BYL719	PI3K	
CCI-779 (Temsirolimus)	mTORC1	
RAD001 (Everolimus)	mTORC1	
Ipilimumab	Anti-CTLA-4	CTLA-4 receptor
